# Habitat use, survival, and migration of a little‐known East Asian endemic, the yellow‐throated bunting *Emberiza elegans*

**DOI:** 10.1002/ece3.10030

**Published:** 2023-05-02

**Authors:** Wieland Heim, Aleksey Antonov, Friederike Kunz, Martha Maria Sander, Marc Bastardot, Ilka Beermann, Ramona Julia Heim, Alexander Thomas, Vera Volkova

**Affiliations:** ^1^ Institute of Landscape Ecology University of Münster Münster Germany; ^2^ University of Turku Turku Finland; ^3^ Swiss Ornithological Institute Sempach Switzerland; ^4^ Khingansky State Nature Reserve Arkhara Russia; ^5^ Independent Researcher Muenster Germany; ^6^ Department of Life Sciences and Systems Biology University of Turin Turin Italy; ^7^ NABU (Naturschutzbund Deutschland) e.V. Berlin Germany; ^8^ University of Lausanne Lausanne Switzerland; ^9^ EuroNatur Foundation Radolfzell Germany; ^10^ Department of Evolutionary Biology and Environmental Studies University of Zurich Zurich Switzerland; ^11^ Independent Researcher Leipzig Germany; ^12^ State budgetary educational institution of additional education of the city of Moscow "Zelenograd Palace of Creativity for Children and Youth" Zelenograd Russia

**Keywords:** color‐ringing, East Asian flyway, forest, full‐cycle ecology, geolocation, migratory connectivity, tracking

## Abstract

Basic information on the ecology of species is key for their conservation. Here we study the ecology of the little‐known yellow‐throated bunting *Emberiza elegans* based on a multi‐year study on its breeding grounds in the Russian Far East. For the first time in this species, we quantified breeding habitat parameters, calculated sex‐specific apparent survival, and determined individual nonbreeding locations using light‐level geolocation. We found that the habitat around song posts of male yellow‐throated buntings is characterized by tree and shrub layers on richly littered moist ground. Habitat use overlaps with co‐occurring Tristram's Buntings *Emberiza tristrami* and Black‐faced Buntings *E. spodocephala*, but territories differ especially in tree cover and litter cover. Based on 4 years of color‐ringing data of 72 individuals, we calculated an apparent survival rate of 36%, with higher survival estimates for male than for female yellow‐throated buntings. We found no effect of carrying a geolocator on survival. We retrieved six geolocators from males. All birds migrated south‐westward during autumn and spent the nonbreeding season at locations in China 700–1700 km away from their breeding sites. At least two individuals spent the boreal winter outside of the known range in northern or central China. Birds left the breeding area between early October and early November and returned between mid‐March and mid‐April. Our data on habitat use, survival rate, and migratory connectivity will help to assess threats to the populations of this enigmatic species, which might include habitat loss due to forest fires on the breeding grounds, and unsustainable harvest for consumption during the nonbreeding season.

## Graphical abstract

Basic information on the ecology of species is crucial for conservation. Here we provide data on breeding habitat use, survival rates, and spatiotemporal distribution for the little‐studied yellow‐throated bunting. We found that individuals breeding in the Russian Far East migrate to China during the boreal winter.

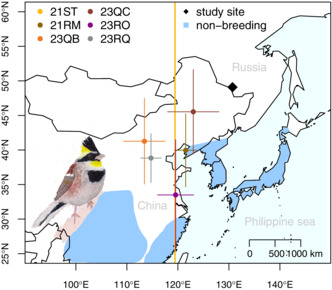

## 1 INTRODUCTION

Understanding the full‐cycle ecology of species is key to their conservation. Knowledge of the spatiotemporal distribution is especially important for migratory species, which rely on multiple sites during the annual cycle (Kirby et al., 2008). Little is known about the migration ecology of many songbird species migrating along the East Asian flyway, but populations are declining in many of those (Yong et al., 2015, 2021). One of those little‐known species is the yellow‐throated bunting (also known as Elegant Bunting) *Emberiza elegans*. Its distribution is confined to East Asia, with breeding populations in the Russian Far East, northern China, and the Korean peninsula (*E. elegans elegans*) as well as eastern China (*E. elegans ticehursti*) (Copete, 2020). In the 1990s, first breeding was also documented in Japan (Ueno et al., 1995). During the breeding season, yellow‐throated buntings prefer young hardwood forests, but rich grassy river valleys are also occupied locally (Dement'ev & Gladkov, 1954). Deciduous and coniferous forests are used as stopover habitats (Averin, 2012; Heim, Eccard, & Bairlein, 2018), but birds can also be found in willow shrubs during migration (Heim, Eccard, & Bairlein, 2018). The southern populations are expected to be sedentary, whereas birds from the northern populations are considered migratory and have been observed during winter in Japan, Taiwan, and southern China (Dement'ev & Gladkov, 1954). Using stable isotopes from feathers collected in South Korea and Japan, a chain migration pattern was suggested for the northern breeding population: birds originating from higher latitudes were found to migrate earlier than birds originating from lower latitudes (Choi, Nam, Park, & Bing, 2020). Birds from northern breeding areas seem to spend the winter farther north than birds originating from breeding areas further south (Choi, Nam, Park, & Bing, 2020). Birds of northern origin have also been shown to have wings adapted to migration, whereas the morphology of birds of southern origin suggests reduced flight efficiency (Zhang et al., 2023). However, details on the migration routes and the connectivity between populations of the yellow‐throated bunting are still unknown due to the lack of tracking studies and the scarcity of ring recoveries (McClure, 1974; Averin, 2012, but see Biodiversity Center of Japan, 2011).

The global population of the yellow‐throated bunting is expected to be stable (BirdLife International, 2022). However, recent studies documented significant population declines in South Korea (Choi, Nam, Kim, et al., 2020; Kim et al., 2021) and Primorye, Russian Far East (Valchuk et al., 2017). Similar to other East Asian *Emberiza* buntings, yellow‐throated buntings might suffer from unsustainable harvests during the nonbreeding season (Heim et al., 2021; Kamp et al., 2015). There is evidence that yellow‐throated buntings are caught and sold for consumption in South Korea (Choi & Nam, 2020), and thousands were found to be traded in a bird market in southern China (Dai & Zhang, 2017). Another possible threat to the species is forest fires that could decrease the available breeding habitat (Averin, 2012). In the species’ core distribution in the Russian Far East, forest fires are expected to increase due to climate change and changes in the water regime (Novorotskii, 2007; Reddy & Sarika, 2012; Yu et al., 2017).

Identifying flyways and investigating migratory connectivity and survival rates will help to assess the effects of known threats (such as harvest) on breeding populations of the yellow‐throated bunting. Furthermore, a detailed understanding of the habitat preferences of the yellow‐throated bunting is important to predict potential population‐level effects in the light of global change (e.g., an increase in fires on the breeding grounds). Here, we compiled different data sets to (1) quantify breeding habitat use, (2) estimate apparent survival and longevity, and (3) describe nonbreeding distribution and migration phenology of a yellow‐throated bunting population breeding in the Amur region, Russian Far East.

## 2 MATERIALS AND METHODS

### 2.1 Study area

Our study was conducted at Khingansky state nature reserve in a river valley near the village of Kundur, Amurskaya oblast, Russia (130.71°E, 49.09°N, Figure 2). The study area is situated in the center of the breeding distribution of nominate yellow‐throated buntings and is covered by mixed broadleaf forest. The region has a temperate climate with features of continentality and is affected by the summer monsoon.

### 2.2 Habitat use

In order to map occupied territories, we searched for singing males during early May. The locations of singing males were marked with handheld GPS devices. We counted all positions as occupied territories if a singing male was present twice within 1 week. Habitat data were collected within a 10 × 10 m plot, with the positions of singing males in the center, similar to earlier studies on the habitat use of other East Asian bunting species (Beermann et al., 2021; Heim, Thomas, et al., 2022). All habitat data were collected between 17 and 20 May 2018. Habitat parameters included total vegetation cover, tree cover, conifer cover, tree height, shrub cover, shrub height, cover of deadwood (snags and logs), graminoid cover, graminoid height, herb cover, herb height, litter cover, litter height, bare soil cover, moisture, and inclination. Heights (shrub/tree height in m, all other in cm) and cover (all in %) of vegetation layers as well as inclination (in %) were estimated by eye by the observers from the corners of the plot after several trial runs and satisfying synchronization in the resulting estimates. Height and percentage cover values below 10 were estimated in increments of one, and those above 10 in increments of five. The cover of vegetation layers was estimated independent of each other due to possible overlap. Soil moisture was estimated on a scale of zero to three, with zero signifying “completely dry,” one signifying “moist,” two signifying “waterlogged,” and three “open water.” Furthermore, we noted whether there were signs of recent fire on the study plot (fire: yes) or not (fire: no), and whether there was a creek or river within 100 m distance of the plot (yes/no).

To compare habitat use with co‐occurring and closely related species, we also searched for territories and collected habitat data as described above for Black‐faced Bunting *Emberiza spodocephala* and Tristram's Bunting *E. tristrami*.

We performed an ordination by including the habitat parameters with a self‐organizing Map (SOM) using the Kohonen package (Wehrens & Buydens, 2007) in R, version 4.1.3 (R Core Team, 2022). We defined an output map of 6 × 6 hexagons and rlen = 1000. We excluded all parameters with NA values and ended up with 14 habitat parameters (total vegetation cover, tree cover, conifer cover, shrub cover, shrub height, cover of deadwood, graminoid cover, graminoid height, herb cover, litter cover, litter height, bare soil cover, moisture, and fire).

The Kohonen SOM is an artificial neural network that learns to show multidimensional input data in a low‐dimensional output map through unsupervised learning (Kohonen, 2012). The SOM consists of two layers of artificial neurons: (1) the input layer, fed by external data (here the table with habitat parameters) and (2) the output layer, a two‐dimensional map out of hexagons, in which each hexagon represents a neuron. Every hexagon is linked to the input layer with a so‐called weight vector (Park et al., 2006).

The SOM algorithm performs several steps to display the occupied territories with habitat parameters of the input data in the output map: (1) The learning algorithm calculates the distance between one random sample unit (in our case one occupied territory) and each hexagon, looking for clusters in the input data (in our case based on habitat parameters). (2) The algorithm chooses the hexagon with the smallest distance to the sample unit as a winner. (3) The information is used to update the weights. These steps are repeated with a random input sample for each run (Giraudel & Lek, 2001).

Until now, SOMs are not widely known and used by ecologists, but they have advantages over other ordination methods (Giraudel & Lek, 2001). SOMs allow, for example, for displaying nonlinear species abundance patterns, which is difficult with traditional linear ordination methods like principal component analysis (Wehrens & Buydens, 2007). Additionally, their result does not depend on the axis included, which is the case using nonmetric multidimensional scaling (Giraudel & Lek, 2001).

### 2.3 Survival

To investigate survival and longevity, we marked birds with one numbered metal ring and an individual combination of three color rings (i.e., two rings on one leg) which enabled identification in the field. The birds were trapped with mist nets (Ecotone, Poland; 6–12 m length, 2.5 m height, 16 mm mesh size) and playback of the song of the species in their territories during the breeding season (May) in 2017, 2018, and 2019. We visited all those territories again in the following years (2018–2020) to search for color‐ringed individuals with the help of playback.

To estimate the survival of individuals, we employed Cormack‐Jolly‐Seber‐Models (CJS) using the package RMark (Laake et al., 2013; White & Cooch, 2001), assuming that individual return rates are a product of individual survival and encounter probability (*p*). As we cannot correct for emigration and mortality, we use the term “apparent survival” (Phi, φ). We modeled both *p* and Phi with unique encounter histories of the birds. The encounter histories included the identifier of the individuals, sex, and whether a bird was encountered in the years 2017–2019 (0 = not encountered in a given year, 1 = encountered, e.g., “1100” = individual ringed in 2017, observed in 2018 but not in 2019 and 2020). Furthermore, we added information on whether a bird was equipped with a data logger or not. We built three models. First, we built a null model to estimate Phi and *p* for all individuals. Second, we employed Phi as a function of “Logger,” a binary variable describing whether an individual was initially equipped with a geolocator or not (males only). Third, we employed Phi as a function of sex to estimate sex‐specific differences in Phi and *p*.

### 2.4 Migration

We equipped 33 adult male yellow‐throated buntings with light‐level geolocators (SOI‐GDL 2.0, Swiss Ornithological Institute) during spring 2018 (*n* = 13) and 2019 (*n* = 20). We used elastic leg‐loop harnesses in spring 2018. However, four out of six recaptured individuals in spring 2019 had lost their tracking devices. Therefore, we used nonelastic leg‐loop harnesses made of 1 mm wide braided nylon strings to attach the devices in 2019. None of the four birds recaptured in spring 2020 had lost its geolocator. The weight of the loggers (0.7 g) corresponded to 3.6–4.2% (mean: 3.8%) of the birds’ body weight at deployment. Capture and recapture were conducted as described above.

Light‐intensity data were recorded at 5 min intervals. Data were corrected for clock drift and analyzed using the GeoLocTools toolbox (Lisovski et al., 2019)in R version 4.1.3 (R Core Team, 2022). Twilights (sunrise and sunset events) were identified on log‐transformed light data and a threshold of 1. We used a breeding site calibration (April–July) to determine the sun elevation angle using the *getElevation* function (Lisovski & Hahn, 2012). The coordinates of stationary periods during migration and the nonbreeding season were calculated using the *coord* function (tol = 0.1–0.2) and timing with the *changeLight* function (quantile = 0.98–0.99) (Lisovski & Hahn, 2012). We only considered stationary sites of more than 3 days due to the very low recorded light levels. Light levels during the year of tracking were plotted using loess smoothing with the *geom_smooth* function in ggplot2 (Wickham, 2016) to visualize changes that could be linked to habitat use or behavior.

## 3 RESULTS

### 3.1 Habitat

**FIGURE 1:**
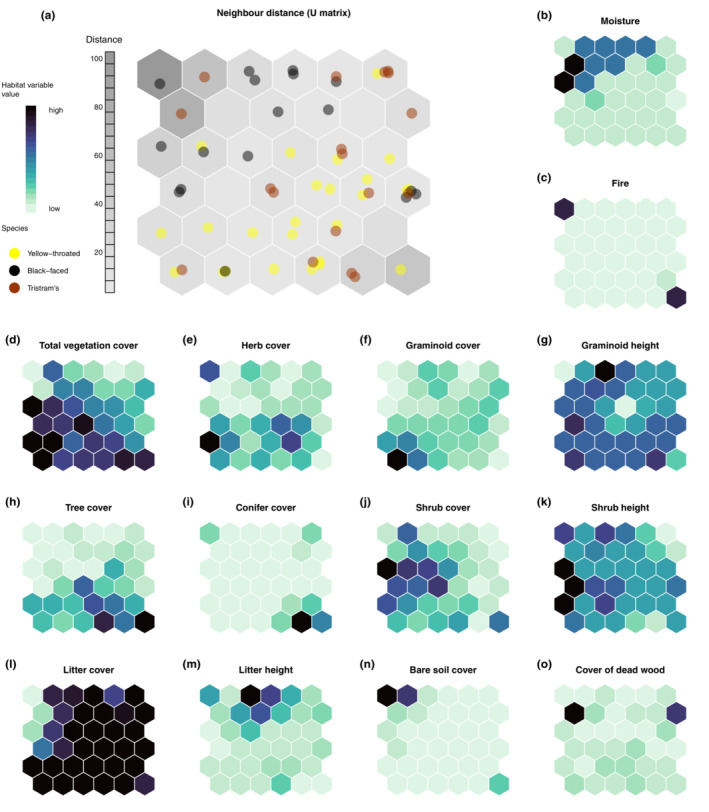
Self‐organizing map (SOM) output neurons (shown as hexagons), displaying the distance between neurons (U‐matrix) in gray colors and assigned territories (a). Territory dots that are clustered within one hexagon are very similar regarding habitat characteristics. The location of the territory dots within the hexagon is random (scattered to avoid overplotting in the center of the hexagon). A long distance from neighbor hexagons is displayed by a dark gray hexagon color, whereas a small distance to neighbor neurons is displayed by light gray. Each hexagon in the output map (a) corresponds to the hexagon in the same location on each of the habitat maps (b–o). For example, territories of the Black‐faced bunting can be found in hexagons in those output neurons in which the habitat‐related map of moisture (b) shows a high probability.

We collected habitat parameters in territories of yellow‐throated buntings (*n* = 25), Black‐faced Buntings (*n* = 17), and Tristram's Buntings (*n* = 18). The distribution of the territories of the three species could be clearly described by habitat parameters (Table 1, Figure 1). The differences in habitat use between species are indicated by a clustering of territories of each species in particular areas of the SOM output map (Figure 1). But the habitats also partially overlapped between species, indicated by the clustering of territories of different species together in the same and nearby hexagons of the SOM output map. This was mainly the case for territories of yellow‐throated bunting and Tristram's Buntings.

**TABLE 1:** Habitat parameters in territories of Yellow‐throated, Black‐faced, and Tristram's Buntings at Khingansky state nature reserve, Russia.

Habitat parameter	Yellow‐throated bunting	Black‐faced bunting	Tristram's bunting
Total vegetation cover (%)	61 (25–90)	42 (12–90)	49 (12–90)
Tree cover (%)	27 (0–80)	5 (0–20)	20 (0–50)
Tree height (m)	14.2 (8–20)	13.0 (5–20)	15.2 (3–20)
Shrub cover (%)	26 (5–65)	28 (3–90)	21 (3–70)
Shrub height (m)	2.1 (1–4)	2.7 (2–4)	1.6 (0–3)
Deadwood (%)	6 (0–15)	2 (0–10)	10 (0–70)
Grass cover (%)	10 (1–45)	6 (0–20)	8 (1–40)
Grass height (cm)	18.0 (5–20)	19.7 (5–30)	17.2 (10–25)
Herb cover (%)	9 (2–30)	6 (0–20)	8 (1–20)
Herb height (cm)	17.8 (10–25)	15.9 (10–25)	16.7 (10–30)
Litter cover (%)	99 (80–100)	83 (20–100)	93 (30–100)
Litter height (cm)	8.0 (1–25)	26.1 (1–100)	8.5 (3–30)
Bare soil (%)	1 (0–15)	6 (0–70)	4 (0–50)
Moisture (0–3)	1.0 (1–2)	1.5 (1–3)	1.2 (0–3)
Inclination (%)	1.2 (0–4)	0.1 (1–2)	1.1 (0–6)
Fire (*n*/*n* total)	1/25	1/17	0/18
Creek (*n*/*n* total)	11/25	15/17	10/18

*Note*: Given are the mean values, with the range in brackets. For an explanation of how the parameters were defined, please see the text.

Territories of the yellow‐throated bunting showed the highest cover values of trees, grasses, herbs, and litter (Table 1). The territories are therefore located in hexagons of the output map, where the habitat‐related maps are showing the highest probability values of these habitat parameters (Figure 1). In contrast, Black‐faced Buntings occupied territories with the lowest total vegetation cover, the lowest tree cover, but a high litter height. Almost all Black‐faced Bunting territories were found close to creeks, with the highest moisture score and lowest inclination. Tristram's Bunting territories were characterized by the tallest trees and the highest cover of dead trees.

### 3.2 Survival

We color‐ringed 72 adult yellow‐throated buntings between 2017 and 2019 (19 females, 53 males), of which we observed 20 returning individuals in the springs of 2018–2020.

The overall survival probability was estimated as Phi = 0.36 ± 0.11, with a probability of encounter of *p* = 0.67 ± 0.23. We found no difference in apparent survival between males carrying a geolocator (Phi = 0.41 ± 0.17) compared to males without a data logger (Phi = 0.39 ± 0.15). Females had a lower apparent survival (Phi = 0.19 ± 0.09) than males (Phi = 0.40 ± 0.13). However, we found higher encounter probability in females (*p* = 1.00 ± 0.00) compared to males (*p* = 0.65 ± 0.24).

Furthermore, we compiled information on longevity of the species based on ringed individuals in our data set. Three individuals reached an age of at least 3 years, all of them males.

### 3.3 Migration

**FIGURE 2:**
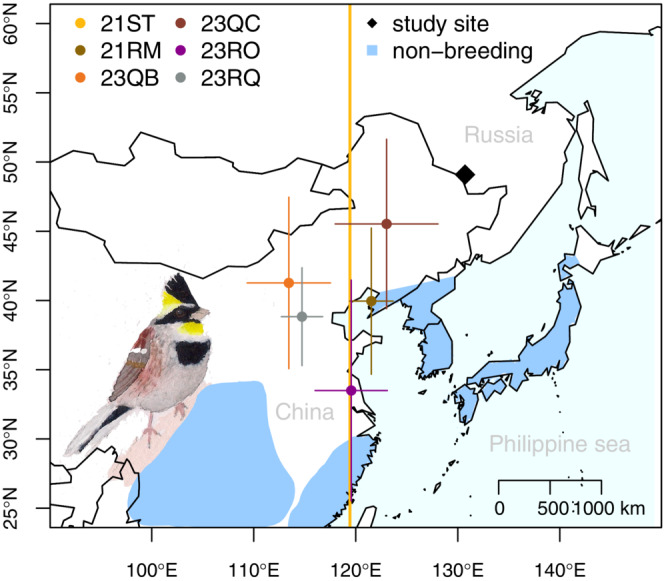
Nonbreeding locations of six individual yellow‐throated buntings breeding at Khingansky state nature reserve, Amur region, Russia based on geolocation data. Nonbreeding range (indicated in blue) following BirdLife International (2022). Note that no latitude could be estimated for individual 21ST, for which a vertical line (in yellow) was plotted to visualize the mean longitude of its nonbreeding location.

We retrieved two geolocators in 2019 and four in 2020. We were able to estimate the coordinates of nonbreeding sites for five individuals, but only the longitude for the sixth individual due to poor light data quality (Figure 2, Table 2). The six individuals spent the nonbreeding season in northern, northeastern, eastern, or central China. The mean distance between breeding and nonbreeding sites was 1366 km (range = 704–1710 km, *n* = 5, Table 2).

**TABLE 2:** Migration timing and nonbreeding locations estimated based on light‐level geolocation data for six individual yellow‐throated buntings breeding at Khingansky state nature reserve, Russia.

Ind.	Departure breeding	Arrival nonbreeding	Departure nonbreeding	Arrival breeding	Dist. (km)	Nonbreeding site (lon, lat)
21ST	2 Oct	3 Oct	15 Apr	16 Apr	NA	119.43°E, NA
23QB	17 Oct	18 Oct	16 Apr	17 Apr	1607	113.43°E, 41.26°N
23RO	19 Oct	20 Oct	21 Mar	21 Mar	1558	117.99°E, 38.47°N
21RM	20 Oct	2 Nov	7 Apr	8 Apr	1249	121.52°E, 39.95°N
23QC	26 Oct	27 Oct	6 Apr	7 Apr	704	123.02°E, 45.52°N
23RQ	4 Nov	5 Nov	16 Mar	17 Mar	1710	114.72°E, 38.83°N

*Note*: Distances are calculated as the great circle distance between the breeding site and the estimated mean coordinates of the nonbreeding site. Individuals are sorted by departure date.

Abbreviations: Ind., individual; Dist., distance; lon, longitude; lat, latitude.

**FIGURE 3:**
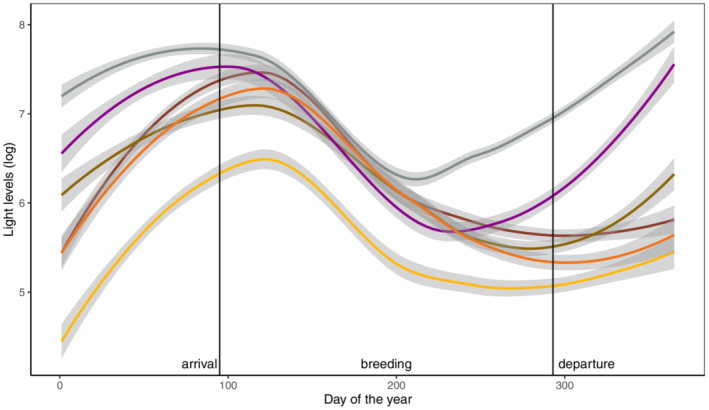
Changes in light levels measured by geolocators mounted on six individual yellow‐throated buntings during the course of 1 year. Note that devices collected data from July until May/June of the following year. Mean arrival/departure dates to/from the breeding site are marked as vertical lines. Colors correspond to individuals as given in Figure 2. Note that loess smoothing was applied to visualize the original data. Gray shading depicts 95% confidence intervals.

On average, birds departed from the breeding site on 19 October (range = 2 October–4 November, Table 2) and arrived in spring on 4 April (range = 17 March–17 April, Table 2). Estimated arrival and departure dates suggest rapid migration between breeding and nonbreeding sites without longer stopovers (Table 2). Overall light levels were very low, especially during the late breeding season and early autumn (Figure 3).

## 4 DISCUSSION

Our results give insights into the ecology of the little‐known yellow‐throated bunting. For the very first time, the habitat use on the breeding grounds and the apparent survival of this species were quantified. Furthermore, we provide the first tracking data of this East Asian endemic.

### 4.1 Habitat use

Yellow‐throated bunting territories were found in open forests with dominant tree and shrub layers on richly littered moist grounds. However, the space used between the three species overlapped for all the investigated parameters. We found the most overlap in habitat use between the yellow‐throated bunting and the Tristram's Bunting, but the latter species seems to prefer older forests with higher trees, less shrubs, and more deadwood. yellow‐throated buntings might therefore prevail also in managed forests. This is confirmed by a study from South Korea, where the numbers of this species increased after forestry‐related thinning of forests (Rhim & Lee, 2001).

Yellow‐throated buntings seem to be less bound to the proximity of wetlands compared to Black‐faced Buntings since only half of the territories were found close to creeks. In our study area, Black‐faced Buntings would mainly occupy territories in shrub‐dominated habitats along creeks and streams, whereas Tristram's and yellow‐throated buntings would occupy territories in forests further away from the water along the slopes of the river valley, as visible, for example, in the inclination data (lowest values for Black‐faced Bunting).

We found one yellow‐throated bunting territory in a recently burned area, suggesting that even forest areas hit by fire can be occupied. Fires are known to be important drivers of habitat availability for other East Asian bunting species that prefer more open habitats (Heim et al., 2019; Heim, Thomas, et al., 2022; Kurdiukov & Volkovskaya‐Kurdiukova, 2016). However, given that fires significantly decrease litter cover (Heim, Heim, Darman, et al., 2020), and that yellow‐throated bunting territories were found in areas with almost 100% litter cover, negative effects of fire on habitat quality can still be expected for this species. Further studies to understand the effects of increasing forest fires on the occurrence and the population size of this species would be required.

Given that we only surveyed habitats around the song posts of males, we cannot make inferences about the habitats required for foraging or nesting. However, for many East Asian bunting species, song posts are known to be very close (10–30 m) to their nesting site (Nakamura et al., 1968).

### 4.2 Survival

We found that 36% of adult yellow‐throated buntings are returning to their territories in the following year. This apparent survival rate is similar or slightly lower compared with other migratory species of comparable size (15–20 g body weight) from temperate latitudes, for example, Spotted Flycatcher *Muscicapa striata* (49%, Siriwardena et al., 1998), Common Redstart *Phoenicurus phoenicurus* (49%, Haukioja, 1969), Whinchat *Saxicola rubetra* (27%, Shitikov et al., 2017), Yellow Wagtail *Motacilla flava* (39%, Shitikov et al., 2017) and Black‐faced Bunting (48%, McClure, 1974). Our survival estimates could be used in a modeling study to understand the effects of potential threats on the population development of this species, as has been done for the closely related Yellow‐breasted Bunting *Emberiza aureola* (Kamp et al., 2015).

We found no effect of geolocator tagging on apparent survival, which could be explained by the rather low relative weight (<4% of body weight for most individuals) of the device for our study species (Brlík et al., 2019). Furthermore, it has to be noted that some individuals tagged with elastic harnesses lost their device—otherwise, their survival probability could have been lower (Brlík et al., 2019).

The lower survival probability estimate for female yellow‐throated buntings could be explained by both lower site faithfulness and higher mortality related to the cost of reproduction, as has been observed in other bird species (Bellebaum & Mädlow, 2015; Grüebler et al., 2008; Pärt, 1995; Székely et al., 2014; Tavecchia et al., 2001). Furthermore, the smaller sample size for females in our study might also skew the results. This is most likely also true for our estimates of encounter probability. The lack of any capture history including a year without observation between years with observations has led to the encounter rate of 100% for females in our study. We consider this highly unrealistic, given the more skulking behavior of females and the fact that female songbirds often respond less towards playback (Herremans, 1989; Schekkerman, 1999; Wojczulanis‐Jakubas et al., 2016). Therefore, a lower encounter rate for females than for males could be expected (Sandercock et al., 2005), which would likely result in an apparent survival estimate similar to that for males.

The oldest yellow‐throated bunting in our study was ringed as a second‐year male in May 2017 and last re‐sighted in May 2019 at the age of 3 years. Data from another station in the Khingansky state nature reserve (129.73°E, 49.40°N) include a recapture of a male at a minimum age of 7 years, further two males with at least 2 and 3 years and one female of at least 2 years (A. Antonov, unpublished data). In a different population in the Russian Far East, adult females were found to return to their previous territories for up to 4 years, and adult males for up to 3 years (Averin, 2012). Overall, longevity in the yellow‐throated bunting seems to be comparable to other migratory *Emberiza* species (e.g., Yellow‐breasted Bunting >7 years, Ortolan Bunting *E. hortulana* > 6 years, Fransson et al., 2010).

### 4.3 Migration

We showed that male yellow‐throated bunting that breed in the Russian Far East are true migrants, with migration distances of up to 1700 km. This contradicts the current assessment of the species by BirdLife International (2022) as “not a migrant.” Other recent studies also demonstrated that northern populations of the species are migratory (Choi, Nam, Kim, et al., 2020; Choi, Nam, Park, & Bing, 2020; Zhang et al., 2023). All individuals migrated south‐westward during autumn to nonbreeding sites on the East Asian mainland, a pattern observed in the majority of migratory East Asian songbird species (Bensch et al., 2022; Heim, Heim, Beermann, et al., 2020; Heim, Pedersen, et al., 2018; Yamaura et al., 2017). An exception to this pattern is the Blue‐and‐white Flycatchers *Cyanoptila cyanomelana* co‐occurring with yellow‐throated buntings at our study site, which were recently found to migrate southward to the Philippines (Heim, Antonov, et al., 2022).

We found that all individuals spent the nonbreeding season in China. This highlights the importance of this country for the conservation of this species. The estimated nonbreeding sites of at least two individuals (23QB and 23RQ) were outside of the known nonbreeding distribution following BirdLife (2022) in northern or central China. Numerous observations from citizen scientists during the boreal winter around Beijing confirm that yellow‐throated buntings are spending the nonbreeding season in this area (e.g., https://ebird.org/species/yetbun1). This could either be a so‐far overlooked nonbreeding area, or birds have only recently shifted their range northward, for example, as a cause of climate change (Lehikoinen et al., 2021). Location estimates provided by us will help to link breeding populations with areas of known (illegal) harvest, as has been demonstrated for the Ortolan Bunting (Jiguet et al., 2019) and Yellow‐breasted Bunting (Heim et al., 2021). Data on the extent of harvest is emerging for many species in Asia (e.g., Gallo‐Cajiao et al., 2020), and future studies should gather such data for our study species and other bunting species that are likely threatened (Heim et al., 2021).

None of the tracked yellow‐throated buntings migrated to Japan. This supports the idea that the considerable nonbreeding population observed in Japan might originate from the southern part of the breeding distribution, as suggested by the stable isotopic assignment of three individuals sampled in southern Japan during the boreal winter (Choi, Nam, Park, & Bing, 2020) and by a bird ringed in southern China and recovered in Japan (Biodiversity Center of Japan, 2011).

We were not able to locate any stopover sites, which could either be caused by low light data quality (especially in autumn, see Figure 3), allowing us to detect only stays longer than 3 days (see Section 2), or by the fact that yellow‐throated buntings migrate indeed without longer stopovers to their nonbreeding destinations. Very short autumn stopover durations (1–3 days) were also observed in adult yellow‐throated buntings at a ringing station in the Russian Far East (Collet & Heim, 2022). Future studies using battery‐powered GPS data loggers might be more promising to understand the species’ migration route in more detail.

The steep decline in measured light levels in late spring at the breeding site is most likely caused by changes in behavior (Lisovski et al., 2012). In early spring, male yellow‐throated buntings would often sing perched on trees, while they do not sing in summer and mainly forage on the ground, where they will experience less light. During the time of molt in late summer/early autumn, they will hide themselves even more, which coincides with the lowest measured light levels. Another explanation for the decline in light levels comes from the change in vegetation (Lisovski et al., 2012), since deciduous trees dominating their breeding habitat will have no leaves in early spring, while the canopy will be closed in summer. Less easy to explain is the increase in light levels during the nonbreeding season. This could be related to defoliation of trees during late autumn, changes in habitat use (a potential switch from forests to more open habitats) or in behavior, with males possibly starting to sing again before they reach their breeding sites. Studies using high‐resolution tracking devices (e.g., GPS data logger) would be necessary to understand whether the observed patterns stem from changes in behavior or habitat use.

The mean arrival/departure dates to/from the breeding site that we have estimated with geolocation confirm previous studies on the species' phenology, rendering it as one of the earliest migrants to arrive and one of the latest to depart in the Russian Far East (Averin, 2012; Dement'ev & Gladkov, 1954; Heim, Eccard, & Bairlein, 2018). Records of ringed individuals have demonstrated that birds stayed close to their breeding sites from April to September (Averin, 2012), but we showed that some males arrived already in mid‐March and stayed until early November.

A major limitation of our study is that we have tracked only adult males, whose spatiotemporal distribution might differ from birds of other ages or sex. First‐year birds have been shown to migrate earlier than adults in autumn, as they will not undergo complete molt, contrary to adults before departure (Averin, 2012; Rashkevich, 2015; Wobker et al., 2021). Male yellow‐throated buntings are known to migrate ahead of females in spring and in autumn (Nam et al., 2011; Wobker et al., 2021), which could point toward differences in the nonbreeding distribution. Future studies with data loggers should therefore also include adult females, although their return rates might be lower, as we have shown above. The use of GPS data loggers could provide spatial information on higher resolution, and such data could be used to infer habitat characteristics of nonbreeding locations.

## AUTHOR CONTRIBUTIONS

**Wieland Heim:** Conceptualization (lead); formal analysis (lead); funding acquisition (lead); investigation (equal); methodology (lead); project administration (equal); software (equal); writing – original draft (lead); writing – review and editing (lead). **Aleksey Antonov:** Conceptualization (supporting); investigation (equal); methodology (supporting); project administration (equal); writing – review and editing (supporting). **Friederike Kunz:** Formal analysis (supporting); investigation (supporting); methodology (supporting); software (equal); writing – review and editing (supporting). **Martha Maria Sander:** Formal analysis (supporting); funding acquisition (supporting); investigation (equal); methodology (supporting); writing – review and editing (supporting). **Marc Bastardot:** Investigation (equal). **Ilka Beermann:** Investigation (supporting); methodology (supporting); project administration (supporting). **Ramona Julia Heim:** Formal analysis (equal); writing – review and editing (supporting). **Alexander Thomas:** Investigation (supporting); methodology (supporting). **Vera Volkova:** Investigation (supporting).

## CONFLICT OF INTEREST STATEMENT

The authors declare that they have no conflict of interest.

## Data Availability

The location data based on solar geolocation presented in the current study are publicly available at Movebank (www.movebank.org) in the study “Yellow‐throated Bunting (Emberiza elegans) Amur/Russia” (Movebank study ID 2711309904). Data on habitat use and survival (capture histories) used in the current study are publicly available on Dryad (https://doi.org/10.5061/dryad.mw6m90620). Open Access funding enabled and organized by Projekt DEAL.
